# Biophilic design in hospital environments: a rapid review of nature-integrated strategies and patient outcomes

**DOI:** 10.3389/fpubh.2026.1786483

**Published:** 2026-03-03

**Authors:** Yishan Du, Mei Xie, Yuanyuan Zou

**Affiliations:** 1 Product and Service Design — PSD, Sapienza University of Rome, Rome, Italy; 2 Department of Psychology of Developmental and Socialization Processes, Sapienza University of Rome, Rome, Italy; 3 Department of Neurosciences, Mental Health and Sensory Organs — NESMOS, Sapienza University of Rome, Rome, Italy

**Keywords:** attention restoration, biophilic design, healing environments, hospital architecture, patient outcomes, stress recovery

## Abstract

Biophilic design has received growing attention as hospitals increasingly seek evidence-based strategies to support patient recovery, psychological comfort, and staff well-being. This rapid review synthesizes empirical findings on nature-integrated design interventions in hospital settings and evaluates their impacts on patient outcomes. Searches across Web of Science, Scopus, PubMed, and Google Scholar did not impose a formal restriction on publication year; however, priority was given to more recent empirical studies in order to capture the latest developments in biophilic design research within healthcare environments. Convergent evidence shows that biophilic interventions—including indoor plants, green views, natural materials, optimized daylight, healing gardens, and nature-themed digital media—enhance stress recovery, reduce pain, improve emotional well-being and sleep quality, and elevate satisfaction with care. Effects are strongest for direct exposure to real nature and moderately strong for simulated nature. Proposed mechanisms include attentional restoration, stress reduction, positive environmental appraisals, and heightened perceived control. However, evidence for long-term clinical outcomes remains limited, and methodological heterogeneity constrains cross-study comparability. Overall, this review highlights promising design strategies and underscores the need for more rigorous longitudinal and interdisciplinary research to advance biophilic healthcare design.

## 1 Introduction

Hospitals are complex and high-stress environments where patients routinely experience pain, uncertainty, anxiety, and reduced autonomy. Over the past decade, research has increasingly emphasized the role of the physical environment in shaping recovery trajectories, emotional well-being, and perceived quality of care (1). Recent architectural research further highlights how biophilic principles embedded in hospital buildings enhance both health and esthetic experience, demonstrating that nature-integrated design can significantly elevate users’ psychological comfort (2). This emphasis is supported by systematic evidence demonstrating the health and well-being benefits of biophilic design in healthcare settings (3) as well as its broader contributions to human well-being across built environments (4).

Biophilic design, defined as an approach that intentionally integrates natural elements, processes, and patterns into architectural and interior environments to support human well-being, has therefore emerged as a promising framework for creating restorative healthcare settings (5, 6), aligning with recent work that conceptualizes biophilic architecture as a holistic and sustainable approach to promoting health and environmental quality (7). In this review, “patient outcomes” refer specifically to four domains: psychological outcomes (e.g., stress, anxiety, mood), physiological indicators (e.g., heart rate, cortisol, sleep quality), clinical and behavioral outcomes (e.g., pain medication use, length of stay, rehabilitation engagement), and experiential outcomes such as satisfaction and perceived quality of care. Recent syntheses have clarified its core parameters and applications in clinical environments (8). The approach is grounded in evolutionary psychology and draws on stress-recovery theory (9) and attention restoration theory (10), both of which posit an inherent human affinity for natural elements. This premise is elaborated in Ulrich’s (11) later theoretical work and reinforced by hospital design scholarship that highlights the therapeutic benefits of nature-based strategies (12), as well as psychological evidence showing that biophilic design consistently improves mood, stress recovery, and overall well-being across built environments (13).

Within hospital contexts, nature-integrated strategies operate across multiple spatial scales—from architectural interventions to interior applications such as indoor plants, natural textures, and aquariums. These approaches not only enhance environmental quality and support healing processes (14–16) but also reflect a well-established body of work demonstrating how biophilic patterns can be effectively translated into practical built-environment design (17). Hospital-focused evaluations further show that these strategies encompass a broad range of biophilic design parameters (18). These strategies have been documented across diverse cultural contexts, including evidence from Libyan hospital interiors demonstrating the application of biophilic elements to enhance environmental quality and patient experience (19, 20). They also include sensory stimuli such as daylight and water soundscapes, as well as digital or immersive simulations like virtual nature and projected landscapes, aligning with recent conceptual work that identifies essential biophilic elements for healing-oriented spaces (21). Evidence from outpatient clinics further demonstrates the psychosocial benefits of biophilic design across diverse healthcare settings, indicating its applicability beyond inpatient environments (22).

Although previous reviews have examined individual components—such as green spaces, daylight, or specific landscape features—and commentaries have underscored the broad therapeutic potential of biophilic design in healthcare environments (23), few have synthesized evidence across the full spectrum of biophilic modalities present within hospitals. Unlike prior reviews that focus on isolated design elements or single outcome categories, the present review synthesizes evidence across the full spectrum of biophilic modalities and explicitly integrates environmental mechanisms, moderators, and outcome domains to provide a more holistic analytic lens. This limitation persists despite broader analyses identifying emerging trends, conceptual gaps, and future research directions for biophilic design in the built environment (24).

Given the rapidly expanding and interdisciplinary nature of research on biophilic design in healthcare environments, a rapid review approach was adopted to provide a timely, evidence-informed synthesis that can inform both research and design practice. The review also examines underlying mechanisms, key moderators, methodological limitations, and directions for future research. While staff well-being is acknowledged as an important related issue, the primary focus of this review is on patient outcomes; staff outcomes are discussed only where they are directly linked to patient care or environmental mechanisms.

## 2 Methods

### 2.1 Review approach

A rapid review methodology was adopted to provide an evidence-informed synthesis within a shorter timeframe while maintaining core principles of systematic searching and transparent reporting. This method is appropriate for emerging interdisciplinary fields such as environmental health and healthcare design.

### 2.2 Search strategy

Searches across Web of Science, Scopus, PubMed, and Google Scholar were conducted without a formal restriction on publication year; however, priority was given to more recent empirical studies to capture current developments in healthcare biophilic design. The search strategy combined terms related to biophilic design (e.g., “biophilic design,” “nature-based design,” “healing environment”), healthcare settings (e.g., “hospital,” “healthcare facility,” “clinical environment”), and patient-related outcomes (e.g., “patient outcomes,” “recovery,” “stress reduction,” “well-being”). Grey literature, including WHO reports and healthcare design guidelines, was screened to identify additional relevant materials, although these sources were not included in the formal analysis.

### 2.3 Inclusion criteria

Studies were eligible for inclusion if they focused on hospital or inpatient clinical settings and examined environmental features consistent with biophilic design principles. Eligible studies were required to report at least one psychological, physiological, behavioral, or clinical patient outcome and to employ quantitative, qualitative, or mixed-method research designs. Studies focusing primarily on staff outcomes were excluded unless patient outcomes constituted the primary analytic focus. Only peer-reviewed articles published in English were included, a decision that is acknowledged as a potential source of language bias. Studies focusing on neonatal intensive care units (NICUs) were excluded due to their highly specialized sensory, technological, and infection-control environments, which limit the applicability of general biophilic design principles.

Study selection was conducted in two stages (title/abstract screening followed by full-text review). Data extraction focused on study design, hospital setting, biophilic intervention type, exposure duration, outcome domains, and key findings. Given the heterogeneity of study designs, a formal risk-of-bias assessment was not conducted; instead, methodological limitations were considered narratively. Findings were synthesized using a narrative thematic approach organized by outcome domain.

Given the substantial methodological heterogeneity across studies—particularly in outcome measures, exposure duration, and operational definitions of biophilic design—a narrative synthesis approach was adopted rather than quantitative aggregation, in line with best practices for rapid reviews in interdisciplinary fields.

## 3 Results

### 3.1 Impacts on psychological outcomes

Across studies, nature-integrated design was consistently associated with reductions in anxiety, stress, and negative affect; however, effects were stronger and more reliable in studies involving direct exposure to real nature compared to simulated or representational forms, indicating a dose–fidelity gradient in psychological benefits. Patients with access to natural views or healing gardens typically demonstrate faster emotional recovery and, in some cases, a reduced need for sedatives or anxiolytics.

Simulated nature—such as virtual forest landscapes or immersive digital environments—also produces moderate reductions in anxiety, as demonstrated in controlled VR experiments showing that biophilic indoor virtual environments can accelerate stress and anxiety recovery (25), although its effects are generally weaker than those of real nature. Nonetheless, recent EEG-based studies show that high-fidelity virtual reality (VR) nature experiences can elicit measurable biophilic responses (26), and immersive virtual hospital room scenarios have been found to enhance perceived healing (27). Neuropsychological research further demonstrates that biophilic indoor environments can evoke restorative effects even among healthy adults (28). These findings align with broader reviews identifying both the therapeutic potential and inherent limitations of virtual environments as tools for biophilic design (29).

### 3.2 Impacts on physiological indicators

Across several controlled and observational studies, biophilic features were found to influence several key physiological indicators relevant to patient recovery. Exposure to natural elements was associated with lower heart rate and improved heart rate variability, reflecting enhanced parasympathetic activity and reduced physiological stress, lower heart rate and improved heart rate variability were reported across multiple studies. Studies also reported small to medium reductions in cortisol levels, suggesting decreased activation of stress-related hormonal pathways. In postoperative contexts, biophilic interventions corresponded with modest reductions in opioid or other pain medication use, indicating improved pain management. Additionally, consistent exposure to daylight improved circadian rhythm regulation and sleep quality, a finding that aligns with critical reviews identifying daylight as a central component of biophilic healthcare design (30).

### 3.3 Impacts on clinical and behavioral outcomes

A range of clinical and behavioral outcomes showed strong or moderate evidence of improvement in response to biophilic design interventions, consistent with recent scoping review evidence showing that exposure to nature can positively influence inpatient length of stay and overall recovery patterns (31). Across studies, patients exposed to natural elements demonstrated shorter lengths of stay in some clinical contexts, although findings were mixed. In addition, biophilic design was associated with improved appetite, enhanced nutritional intake, reduced agitation—particularly among individuals with dementia—and higher adherence to rehabilitation tasks. Together, these findings suggest that biophilic design may facilitate recovery indirectly through stress reduction rather than exerting uniform effects on clinical endpoints. These outcomes align with evidence indicating that biophilic architectural strategies can enhance both physical and mental health indicators in hospital settings (32) and with findings identifying specific biophilic design features that directly influence patient health (33). Additional research in orthopedic environments suggests that biophilic features may accelerate recovery trajectories and further support engagement in physical rehabilitation activities (34).

Nature-based distractions—such as views of greenery, water features, or nature-themed displays—were also shown to reduce perceived waiting time and improve procedural cooperation among patients undergoing injections, chemotherapy, or dialysis, contributing to smoother clinical workflows and greater patient comfort.

### 3.4 Impacts on satisfaction and perceived quality of care

Across the majority of studies, exposure to natural elements was associated with higher hospital environment satisfaction, a finding supported by evidence from Malaysian private hospitals demonstrating positive end-user responses to biophilic design implementation (35). Biophilic interventions in oncology settings have likewise been shown to enhance patients’ psychological satisfaction with the care environment (36).

Nature-integrated design features also contributed to improved perceptions of cleanliness, comfort, and overall environmental quality, consistent with evidence showing that biophilic therapeutic spaces are positively evaluated by staff, patients, and family companions (37, 38), and further supported by findings that nature imagery can significantly enhance satisfaction among medically complex rehabilitation patients (39). In several studies, patients reported greater trust in caregivers and provided higher overall experience ratings when natural light, green views, or restorative landscape elements were present, with daylight and nature views emerging as some of the strongest predictors of satisfaction and perceived quality of care.

### 3.5 Environmental mechanisms

Across the included studies, four consistent mechanisms were identified through which biophilic design influences patient outcomes, including stress recovery, attention restoration, perceived control, and positive distraction. First, stress recovery emerged as a core pathway, with natural elements facilitating the rapid downregulation of autonomic arousal. Second, attention restoration played a significant role, as “soft fascination” elicited by natural stimuli helped replenish cognitive resources and improve mental clarity. Third, perceived control was enhanced when patients had access to gardens, windows, or other nature-related affordances, which reduced feelings of helplessness and loss of autonomy. Fourth, positive distraction occurred when nature images, views, or sounds redirected attention away from pain, fear, or procedural discomfort. In addition to these primary mechanisms, environmental appraisal—including judgments that an environment is pleasant, safe, or restorative—was found to partially mediate the effects of biophilic features on a range of psychological and clinical outcomes.

### 3.6 Moderators

Several factors moderated the effects of biophilic design across studies. The type of nature played a significant role, with real nature demonstrating the strongest effects, followed by simulated environments, and then abstract nature imagery. Patient category also influenced outcomes, with effects particularly robust for surgical, oncology, geriatric, and dementia populations, consistent with evidence showing that biophilic strategies in long-term care environments for persons with dementia can significantly enhance environmental support and behavioral stability (40). Additional evidence suggest that age-specific biophilic applications can enhance environmental support for children in pediatric hospital settings (41). Exposure duration was another key moderator, as cumulative or prolonged exposure to nature yielded greater benefits than brief or incidental encounters. Cultural context further shaped the impact of biophilic design, with stronger effects observed in East Asian and Scandinavian studies; notably, research from Chinese healthcare environments has demonstrated substantial benefits of nature-integrated design (42).

## 4 Discussion

**Figure 1:**
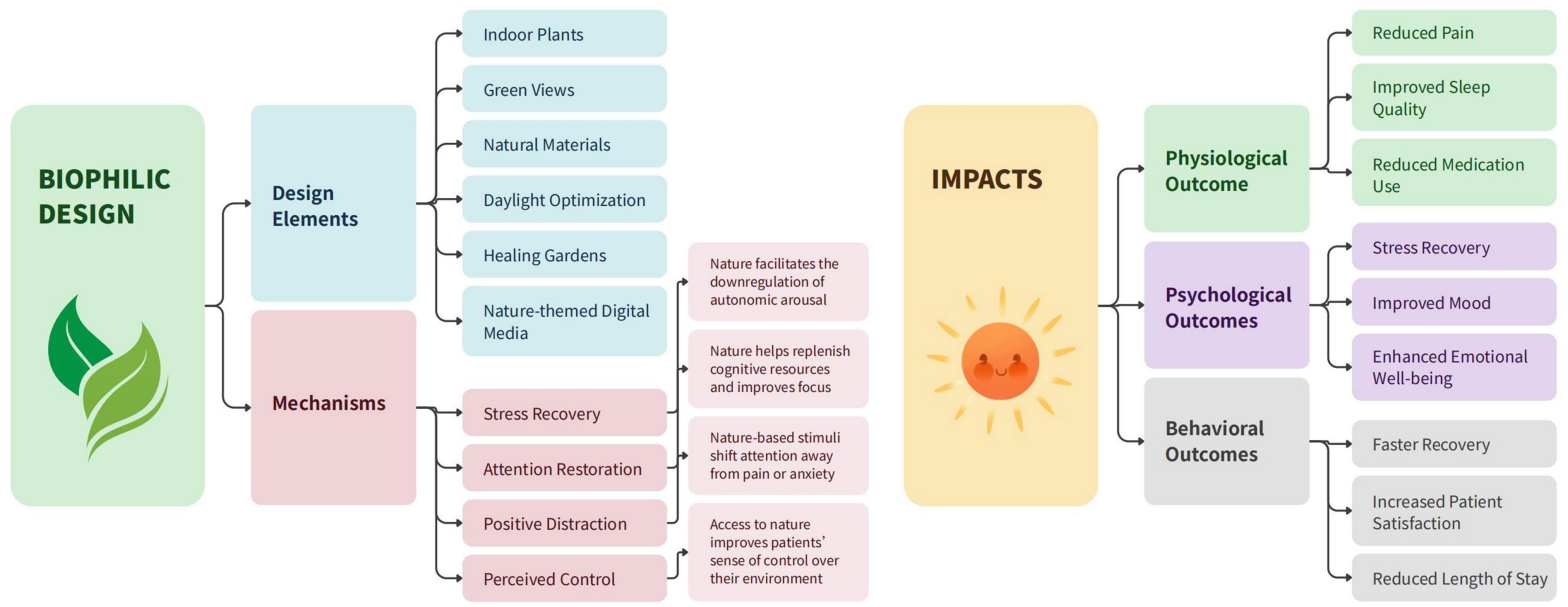
Conceptual framework of biophilic design strategies and patient outcomes in hospital environments.

This rapid review demonstrates that biophilic design is not merely an architectural trend but a consistently effective approach for enhancing patient well-being in hospital environments, as illustrated in Figure 1, which summarizes the pathways linking biophilic strategies to psychological, physiological, and clinical patient outcomes, supported by emerging biophilic frameworks that outline specific design principles for creating healing-oriented interior environments in healthcare facilities (43). This broader therapeutic potential is consistent with emerging work in neuroarchitecture and therapeutic home environments, where intentionally designed biophilic spaces have been shown to support psychological recovery and even enhance digital health outcomes (44). Its benefits have been documented across diverse cultural and clinical contexts, including specialized settings such as cancer care facilities (45) and women’s hospitals in Lagos, where healing-oriented architectural design has shown strong therapeutic value (46). The evidence supports a multi-modal understanding of nature exposure that spans real vegetation, natural materials, sensory stimuli, and visual or digital simulations, aligning with recent interior frameworks that emphasize biophilic elements as essential components of healing-oriented healthcare environments (47).

Real nature—such as gardens, water features, and green views—consistently produces the strongest psychological and physiological effects, a pattern supported both by evidence from hospital resort environments showing substantial mental health benefits from immersive natural exposure (48) and by evaluations of Outdoor Care Retreats demonstrating the high restorative potential of outdoor healing spaces (49). These findings echo meta-synthesis analyses from Maggie’s Centers, which document the profound restorative impacts of nature-integrated architectural features (50). This pattern is also consistent with classic theoretical models suggesting that immersive and multisensory interactions with nature promote both stress recovery and attentional restoration. Indoor plant installations and natural materials contribute additional benefits, supporting restorative design frameworks that integrate biophilia with salutogenesis (51–53), consistent with BaSE (Biophilia–Salutogenesis–Eudaimonia) healthcare design models that emphasize natural, healthy, and emotionally fulfilling environments in hospital architecture (54). Recent work highlights creative strategies for applying biophilic criteria to inpatient room design (55).

Nature-integrated design features also contributed to improved perceptions of cleanliness, comfort, and overall environmental quality, consistent with findings showing that biophilic therapeutic spaces are positively evaluated by staff, patients, and family companions (37, 38), and further supported by evidence that nature imagery can significantly enhance satisfaction among medically complex rehabilitation patients (39). A key insight across the literature is that the effectiveness of biophilic design depends on its interaction with architectural affordances such as privacy, daylight access, acoustic control, and view quality. Biophilic interventions are therefore most impactful when integrated into a holistic healing environment strategy rather than applied as isolated decorative elements, consistent with holistic biophilic design frameworks that emphasize comprehensive, system-level planning to maximize health and well-being outcomes (56). This principle aligns with human-centered, system-level frameworks for healthcare environments (57) and broader design scholarship emphasizing integrated biophilic systems to support restorative outcomes in complex built environments (58).

Despite the promising evidence, several limitations persist, including infrastructure and implementation challenges in developing countries—where cost, policy, and technical constraints hinder the integration of biophilic principles into new hospital construction (59)—as well as the substantial structural, financial, and regulatory adjustments required to retrofit older hospital buildings (60). Research on long-term clinical outcomes—such as readmission patterns or chronic pain trajectories—remains scarce. Few studies employ longitudinal designs or systematically examine staff outcomes, even though existing evidence indicates that biophilic environments can enhance worker efficiency and performance (61). Moreover, staff well-being represents a likely indirect pathway through which environmental design influences patient care quality. Recent work in hospice settings shows that biophilic design can improve emotional well-being, green satisfaction, and workplace attachment among healthcare professionals (62), and biophilic applications in ICU contexts have been proposed as strategies for reducing nurse stress and burnout (63).

## 5 Practical implications for hospital design

### 5.1 Architectural design

While not all biophilic strategies have been empirically tested in every hospital context, the following recommendations reflect convergent empirical evidence and best-practice design principles in healthcare environments. Architectural strategies should incorporate healing gardens, accessible terraces, and courtyards that provide patients with opportunities for direct contact with nature. Such spaces can be designed with diverse vegetation, shaded seating areas, and water features to encourage gentle movement, social interaction, and psychological restoration. Increasingly, hospitals are integrating semi-open therapeutic landscapes that remain usable throughout different seasons, ensuring year-round access even for vulnerable populations.

Patient rooms should be carefully designed to optimize views of natural landscapes from the bedside, ensuring that restorative visual access is available even to individuals with limited mobility or those confined for long periods. Orientation strategies—such as prioritizing patient beds toward window views, reducing visual clutter, and framing natural scenery—can enhance perceived spaciousness and promote a sense of calm.

In addition, the use of natural materials—particularly wood, stone, and textured plant-based panels—can enhance environmental warmth, comfort, and esthetic quality, thereby contributing to a more supportive healing atmosphere. Architectural palettes that mimic natural color gradients or incorporate organic forms may further strengthen the biophilic effect. Importantly, such design strategies should be implemented early in the architectural planning process to ensure compatibility with structural requirements, energy efficiency goals, and infection-control standards.

### 5.2 Interior and wayfinding design

Interior design should include the installation of indoor plants selected from low-allergen species to ensure safety while enhancing environmental vitality. Strategically placing vegetation in waiting areas, corridors, and recovery lounges can soften the clinical atmosphere and reduce perceived stress among patients and families. Green walls or modular planter systems may be integrated into high-traffic areas to create visually engaging focal points without obstructing workflow or maintenance routines.

Nature imagery in corridors, waiting rooms, and transitional spaces can serve as a positive distraction and improve the overall sensory experience of the hospital environment. Research shows that images depicting forests, oceans, or botanical patterns can evoke restorative responses and reduce anxiety before medical procedures. These visual elements can be paired with natural textures—such as woodgrain finishes or stone-like surfaces—to reinforce the continuity of biophilic cues throughout the interior environment.

Furthermore, wayfinding and spatial orientation can be strengthened by integrating natural daylight through skylights, enlarged windows, and other architectural openings that increase luminance levels and support circadian regulation. Employing light gradients, color-coded zones inspired by natural environments, or acoustic treatments that mimic outdoor soundscapes can help patients and visitors navigate the hospital more intuitively. Clear visual hierarchies and biophilic landmarks—such as indoor gardens or nature-themed murals—also reduce wayfinding stress and promote a sense of coherence within large hospital campuses.

### 5.3 Technological solutions

Technological interventions offer valuable alternatives in hospital units where access to real nature is limited. Virtual reality (VR) nature experiences can be deployed for bed-bound patients to provide immersive restorative environments that support stress reduction, emotional regulation, and perceived healing. VR systems can simulate a wide range of natural settings—such as forests, beaches, mountains, or gardens—allowing patients to personalize their preferred environments and enhancing the therapeutic effect through patient-centered choice.

Digital aquariums, projected landscapes, and other nature-themed media installations may also be used in high-stress units—such as intensive care, oncology, or emergency departments—to create calming sensory stimuli and reduce environmental monotony. These systems are particularly valuable in windowless rooms or older facilities where structural constraints limit architectural renovation. Dynamic visual content that changes according to time of day or season can further reinforce circadian cues and create a sense of temporal orientation for long-stay patients.

Such technologies allow hospitals to simulate key psychological benefits of natural environments while accommodating infection-control, spatial, and resource constraints. Furthermore, integrating gentle nature sounds, ambient lighting that mimics sunrise or sunset patterns, or interactive digital murals can create multisensory therapeutic experiences. As digital biophilic technologies continue to advance, hospitals may increasingly adopt hybrid designs that blend architectural nature exposure with personalized technological tools to enhance comfort, resilience, and emotional well-being among diverse patient groups.

### 5.4 Clinical practice integration

Integrating biophilic principles into everyday clinical routines can enhance both patient and staff well-being, transforming biophilic design from a purely architectural intervention into an active component of care delivery. Beyond shaping the physical environment, clinicians and hospital administrators can intentionally incorporate nature-based practices into routine workflows and therapeutic protocols to ensure that patients experience continuous and meaningful engagement with restorative elements.

Providing scheduled or on-demand nature-access breaks—whether through visits to healing gardens, exposure to daylight-rich lounges, access to interior courtyards, or brief supervised outdoor walks—can support emotional recovery, lower stress arousal, and help maintain circadian stability for hospitalized patients. Such practices are especially beneficial for individuals experiencing lengthy stays, high levels of anxiety, or treatment-related fatigue. For healthcare workers, structured nature breaks have been shown to reduce occupational strain and burnout, support emotional regulation during demanding shifts, and improve overall resilience. Incorporating these micro-restorative moments into shift planning or patient schedules helps normalize nature exposure as part of standard care.

In addition, environmental comfort should be incorporated into routine care assessments, enabling clinicians to monitor factors such as lighting adequacy, noise levels, thermal comfort, and access to nature-based features as part of holistic patient management. Small adjustments—such as repositioning beds toward windows, optimizing daylight exposure during waking hours, opening blinds to reveal green views, or introducing nature-based digital media during high-anxiety periods—can meaningfully support physiological regulation and psychological stability. Embedding environmental checks into nursing assessments or patient comfort rounds ensures that the therapeutic potential of the environment is considered alongside medical interventions.

Integrating biophilic practices into clinical routines also requires interdisciplinary collaboration. Nurses, rehabilitation therapists, psychologists, and facility managers can jointly identify areas where biophilic elements support treatment goals—for example, using garden spaces for physiotherapy sessions, conducting mindfulness or counseling outdoors when appropriate, or employing nature imagery to calm patients before invasive procedures. Training staff to recognize the restorative value of natural elements and encouraging them to incorporate these features intentionally enhances consistency in practice.

Embedding these nature-based strategies into standard clinical and operational protocols reinforces the therapeutic role of the physical environment and ensures that biophilic benefits are consistently delivered throughout the care experience. When supported by leadership, included in clinical guidelines, and integrated into broader well-being initiatives, biophilic practices become not only environmental enhancements but also evidence-based components of patient-centered and staff-supportive healthcare delivery.

## 6 Limitations

This rapid review provides a timely synthesis of current evidence, but several limitations should be acknowledged. First, although systematic procedures were followed, the rapid review format constrained the breadth of the search, and some forms of grey literature may not have been comprehensively captured. Second, given the substantial methodological heterogeneity across studies—particularly in outcome measures, exposure duration, and operational definitions of biophilic design—a narrative synthesis approach was adopted rather than quantitative aggregation, in line with best practices for rapid reviews in interdisciplinary fields. Third, most included studies rely on short-term or cross-sectional designs, resulting in a lack of longitudinal evidence on sustained clinical or psychological outcomes. Fourth, the literature remains geographically concentrated, with a disproportionate number of studies conducted in high-income countries, raising concerns about cultural bias and limited generalizability. Fifth, publication bias may also be present, as studies reporting positive outcomes are more likely to be published and therefore more likely to be represented in this review.

To advance the field, future research should adopt mixed-methods or longitudinal designs, harmonize environmental measurement tools, and examine long-term patient trajectories across diverse cultural and clinical contexts.

## 7 Conclusion

Biophilic design significantly contributes to creating healing hospital environments that support patient recovery and psychological well-being. Real nature and daylight exposure have the strongest effects, while simulated nature provides an accessible supplement. As healthcare systems increasingly prioritize patient-centered care and evidence-based design, biophilic design offers low-risk, high-benefit interventions. Continued interdisciplinary research is needed to refine design frameworks, quantify long-term health outcomes, and develop culturally adaptive solutions.
